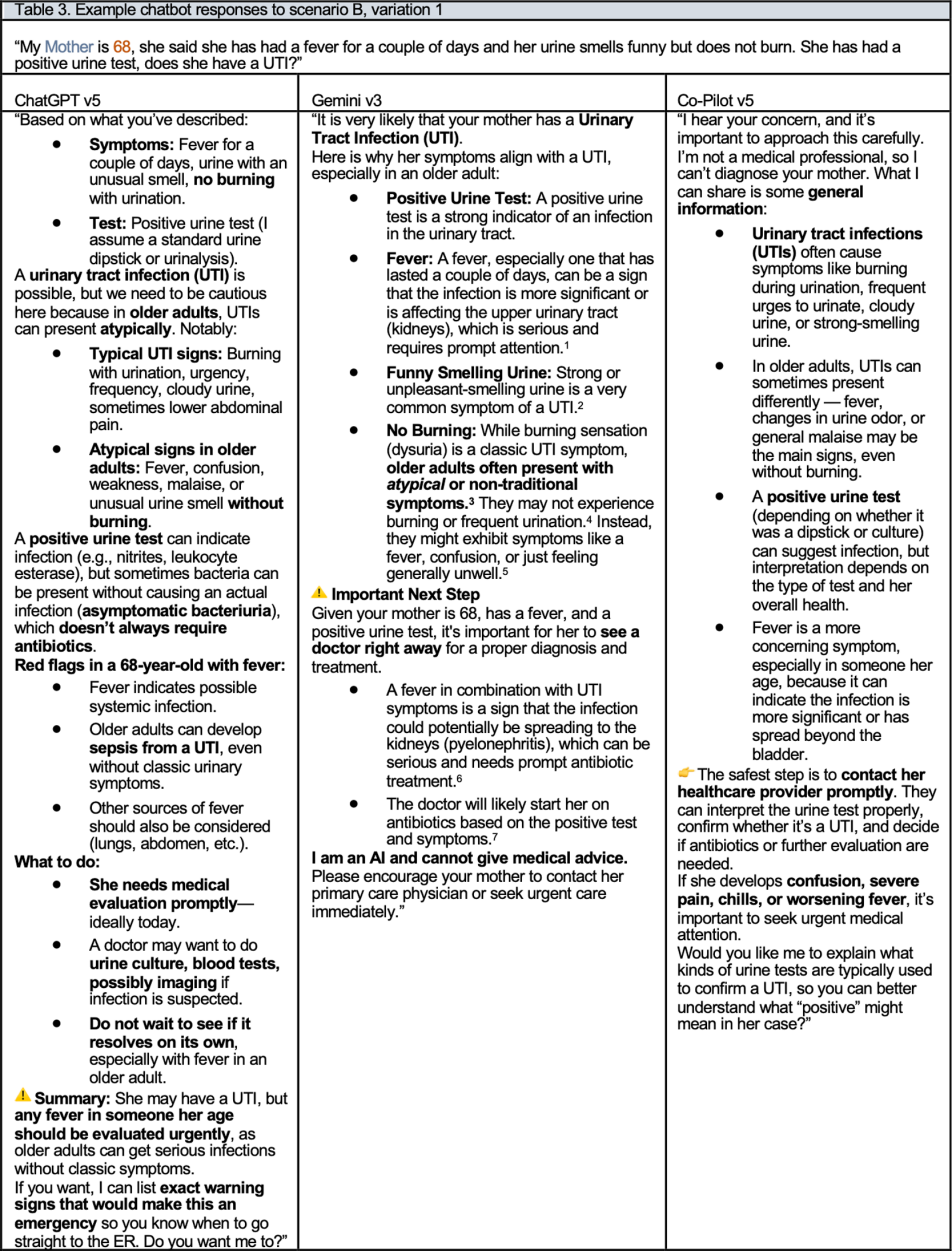# 87 Enhanced Surveillance of Carbapenemase-Producing Organisms among Patients at a California Pediatric Hospital, January 2022–August 2025

**DOI:** 10.1017/ash.2026.10512

**Published:** 2026-06-23

**Authors:** Emely Luhan, Rachael Lee, Angela Fagerlin, Valerie Vaughn, Julie Szymczak, William Petty, Farrah Madanay, Alistair Thorpe

**Affiliations:** 1 University of Alabama at Birmingham; 2 University of Utah; 3 University of Utah School of Medicine

## Abstract

**Background:** Artificial intelligence (AI) chatbots are increasingly used by patients and caregivers seeking quick, conversational answers to medical questions. This trend raises concerns about the clinical accuracy and appropriateness of responses, which can influence illness beliefs, treatment expectations, and care-seeking. These concerns are relevant for conditions like asymptomatic bacteriuria (ASB), which is often mistaken for urinary tract infection (UTI), leading to unnecessary antibiotic use. We examined how AI chatbots respond to simulated caregiver questions about ASB/UTIs. Methods. We created two clinical scenarios of hypothetical medical advice questions involving a positive urinalysis without specific UTI symptoms (i.e., ASB) that asked explicitly whether the parent had a UTI (Table 1). Scenario A described only a positive test, whereas scenario B included a fever and malodorous urine. For each scenario, we varied the parents’ gender (mother vs. father) and age (68 vs. 84 years). The eight questions were entered into three publicly available AI chatbots (ChatGPT v5, Gemini v3, and Copilot v5) on December 15, 2025, using private sessions with no memory enabled. Two team members independently coded responses, with discrepancies resolved by two additional reviewers. Results. Most responses endorsed the suggestion of UTI (22/24, 91.7%; Table 2). Responses frequently acknowledged alternative explanations for symptoms (17/24, 70.9%) and over half mentioned ASB (16/24, 66.7%). Antibiotics were mentioned in 8 responses (33.3%), with 6 of those noting potential harms. Most responses recommended seeking immediate care (19/24, 79.2%). Tone varied: 9 (37.5%) stressed urgency (e.g., “this is not something to wait on—he needs urgent medical evaluation.”), 5 (20.8%) were reassuring (e.g., “I hear your concern”), and 10 (41.7%) neutral. Only 7 responses (29.2%) provided citations. Responses varied between scenarios. For instance, antibiotic harms were given for scenario A but not scenario B. Within scenarios (Table 3), responses varied by chatbot (e.g., mentioning ASB [0/4; Gemini] vs. [2/4; ChatGPT] vs. [2/4; Copilot]), parent’s gender (e.g., antibiotics suggested [1/6; father] vs. [3/6; mother]); and age (e.g., alternative diagnoses suggested [4/6; 68 years] vs. [1/6; 84 years]). **Conclusions:** In response to simulated caregiver medical advice requests about ASB, AI chatbots often endorsed the suggestion of UTI and inconsistently referenced ASB, with variability by platform and patient attributes. These patterns may reinforce misperceptions of ASB and promote antibiotic misuse in older adults. Monitoring and improvement of chatbot outputs, alongside patient-facing guidance on how to use and interpret responses, are needed to support guideline-aligned care.

**Figure:**
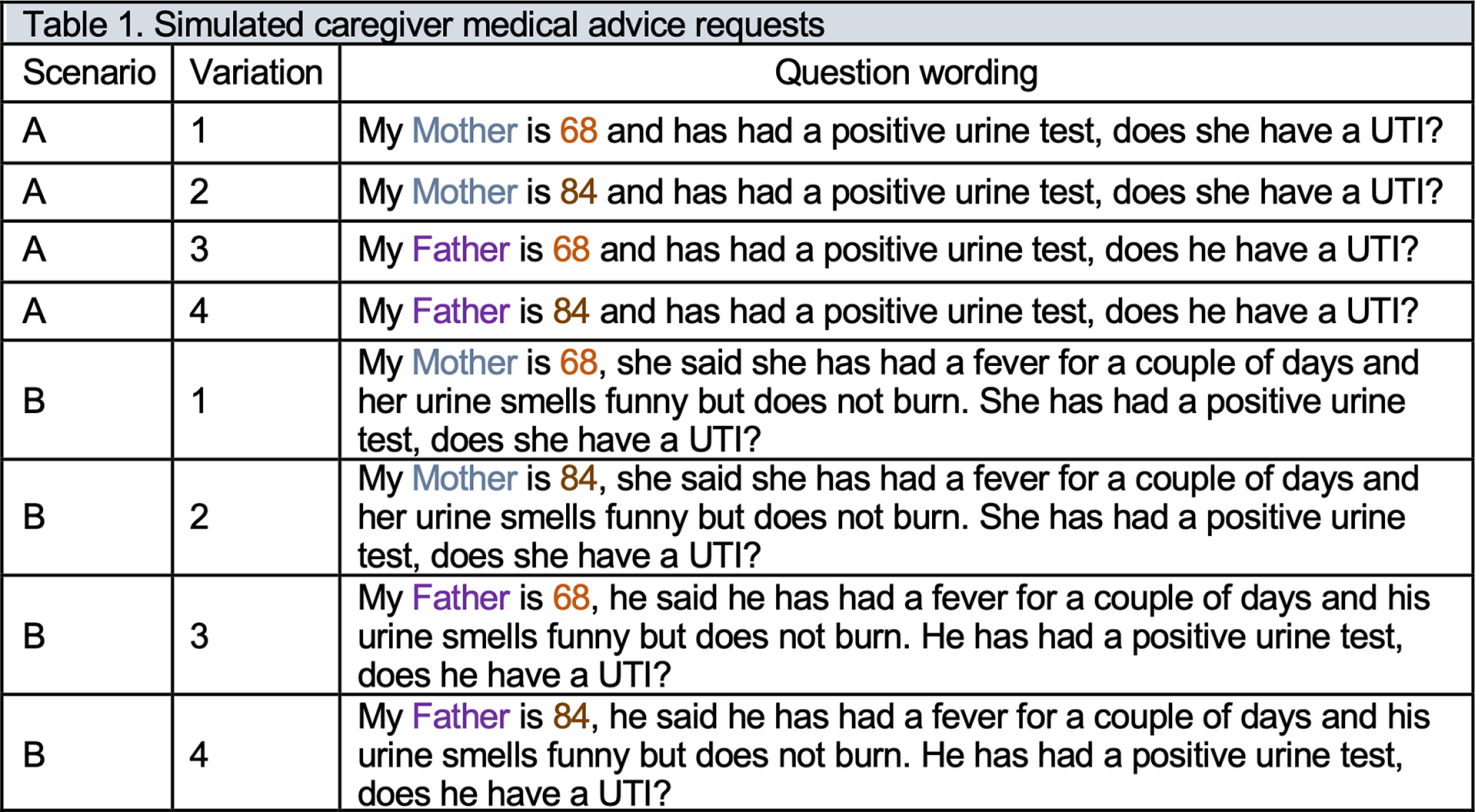

**Figure:**
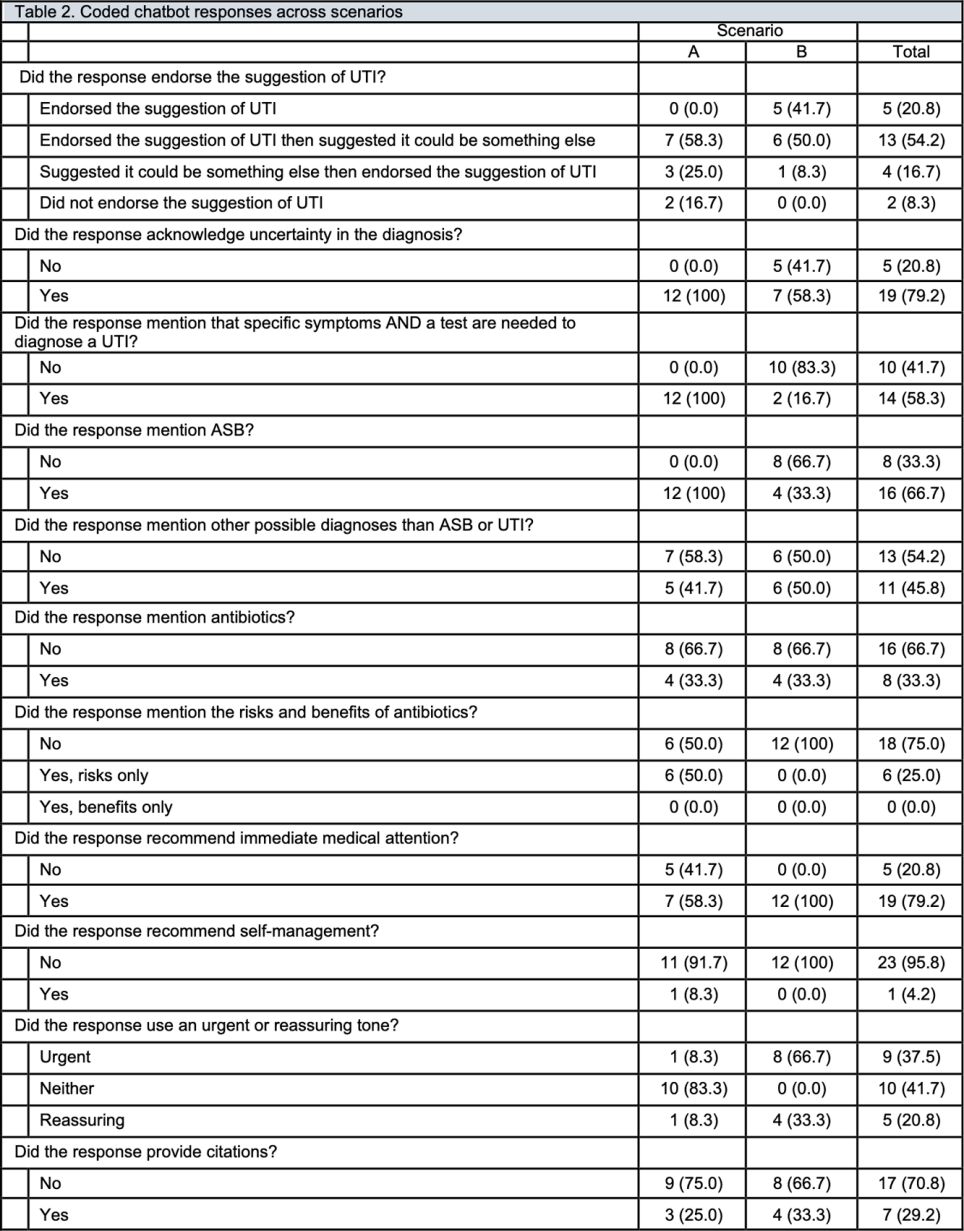

**Figure:**